# **Synergistic effect of Pseudomonas putida and endomycorrhizal inoculation on the physiological response of onion** (*Allium cepa* L.) **to saline conditions**

**DOI:** 10.1038/s41598-024-71165-0

**Published:** 2024-09-13

**Authors:** Mona S. Abd El-Aal, Hanaa R. M. Farag, Ola H. Abd Elbar, Mona S. Zayed, Gamal S. Khalifa, Yasmin M. R. Abdellatif

**Affiliations:** 1 Agricultural Botany Department, Faculty of Agriculture, Ain Shams University, Cairo, Egypt; 2 Biochemistry Department, Faculty of Agriculture, Ain Shams University, Cairo, Egypt; 3 Agricultural Microbiology Department, Faculty of Agriculture, Ain Shams University, Cairo, Egypt

**Keywords:** Salinity stress, *Allium cepa*, Plant growth-promoting rhizobacteria, *Pseudomonas putida*, Endomycorrhizal fungi, Biotechnology, Plant sciences

## Abstract

Salinity stress negatively affects the growth and yield of crops worldwide. Onion (*Allium cepa* L.) is moderately sensitive to salinity. Beneficial microorganisms can potentially confer salinity tolerance. This study investigated the effects of endomycorrhizal fungi (M), *Pseudomonas putida* (Ps) and their combination (MPs) on onion growth under control (0 ppm), moderate (2000 ppm) and high (4000 ppm) NaCl salinity levels. A pot experiment was conducted with sandy loam soil and onion cultivar Giza 20. Results showed that salinity reduced growth attributes, leaf pigments, biomass and bulb yield while increasing oxidative stress markers. However, individual or combined inoculations significantly increased plant height, bulb diameter and biomass production compared to uninoculated plants under saline conditions. MPs treatment provided the highest stimulation, followed by Pseudomonas and mycorrhizae alone. Overall, dual microbial inoculation showed synergistic interaction, conferring maximum benefits for onion growth, bulbing through integrated physiological and biochemical processes under salinity. Bulb yield showed 3.5, 36 and 83% increase over control at 0, 2000 and 4000 ppm salinity, respectively. In conclusion, combined application of mycorrhizal-Pseudomonas inoculations (MPs) effectively mitigate salinity stress. This approach serves as a promising biotechnology for ensuring sustainable onion productivity under saline conditions.

## Introduction

Salinity is a major abiotic stressor affecting crop production globally, including in arid regions like Egypt. High salinity induces osmotic and ionic stress in plants, resulting in inhibited growth and reduced yields^1^. Onion (*Allium cepa* L.) is an economically important vegetable crop, and it is classified as moderately sensitive to salinity^2^. Salinity can reduce onion yields by 18.52% per unit increase in soil salinity (measured in dS/m) above the crop’s threshold which is 1.4 dS/m^3^_._ Salt stress triggers physiological and biochemical alterations in onions, causing osmotic and ionic imbalances, oxidative stress, and nutrient deficiencies. These factors significantly hinder growth and diminish both the quantity and quality of bulb yield, reduced water status, membrane damage, impaired photosynthesis and altered antioxidant systems^4^. Therefore, developing strategies to improve salinity tolerance in onion is critical to ensure sustainable production.

*Pseudomonas* spp. are well-studied as plant growth-promoting rhizobacteria (PGPR) that have been shown to improve plant growth and stress tolerance through direct and indirect^5^. The direct mechanisms include nitrogen fixation, phosphate solubilization, iron chelation and phytohormone production, while indirect mechanisms involve induced systemic tolerance mediated by the accumulation of osmolytes, antioxidants and defense enzymes^6^. *Pseudomonas* spp. exhibited induced systemic resistance against salinity in several crops like wheat, rice and maize^7^. Moreover, recent studies have shown that *Pseudomonas* inoculation can enhance the activity of antioxidant enzymes such as superoxide dismutase (SOD), catalase (CAT), and peroxidase (POD) in salt-stressed plants, contributing to improved ROS detoxification and membrane integrity . The ability of Pseudomonas to produce 1-aminocyclopropane-1-carboxylate (ACC) deaminase, which reduces ethylene levels in stressed plants, has also been highlighted as a key mechanism in alleviating salt stress effects^8^.

In the same context, various researchers have reported the use of endomycorrhizal fungi in alleviating adverse effects of salinity in onions and enhancing plant biomass and bulb yield^9^, due to their ability to produce metabolites, biostimulants, and signaling molecules that trigger plant defenses and enhance systemic toleranc^10,11^. Also, endomycorrhizal fungi, can improve nutrient and water absorption, and also confer abiotic stress tolerance through various morphological, physiological and biochemical changes^12^. Moreover, they mitigate osmotic and oxidative damage in plants under salinity by regulating ion homeostasis, osmolyte accumulation and antioxidant systems^13,14^.

The use of PGPR and endomycorrhizal fungi are promising biotechnological approaches to enhance stress resistance in crops^15,16^. Various researchers have reported that the combined application of PGPR and endomycorrhizae can reveal various synergistic effects on plant salt tolerance. Co-inoculation of *Pseudomonas* spp. and *Glomus* species significantly improved growth, nutrient uptake and antioxidant activity in carrots under saline conditions compared to single inoculations^17^. Also, *Pseudomonas* spp. can stimulate mycorrhizal functioning by improving root colonization and increasing fungal growth mass^18^. *Pseudomonas* and endomycorrhizal fungi represent promising tools to improve salinity tolerance in onion plants grown in arid regions, where soil salinization poses major limitations to agriculture.

The present study hypothesized that co-inoculation with *Pseudomonas putida* and mycorrhizal fungi as biocontrol agents will have a synergistic effect in improving the salt tolerance of onion plants. Our research focused on illustating the physiological mechanisms of *Pseudomonas putida*, and endomycorrhizae which were applied individually or in combination to enhance onion plant tolerance to salt stress and improve bulb yield.

## Results

### Infection percentage of biological agents in the onion root

**Fig.1:**
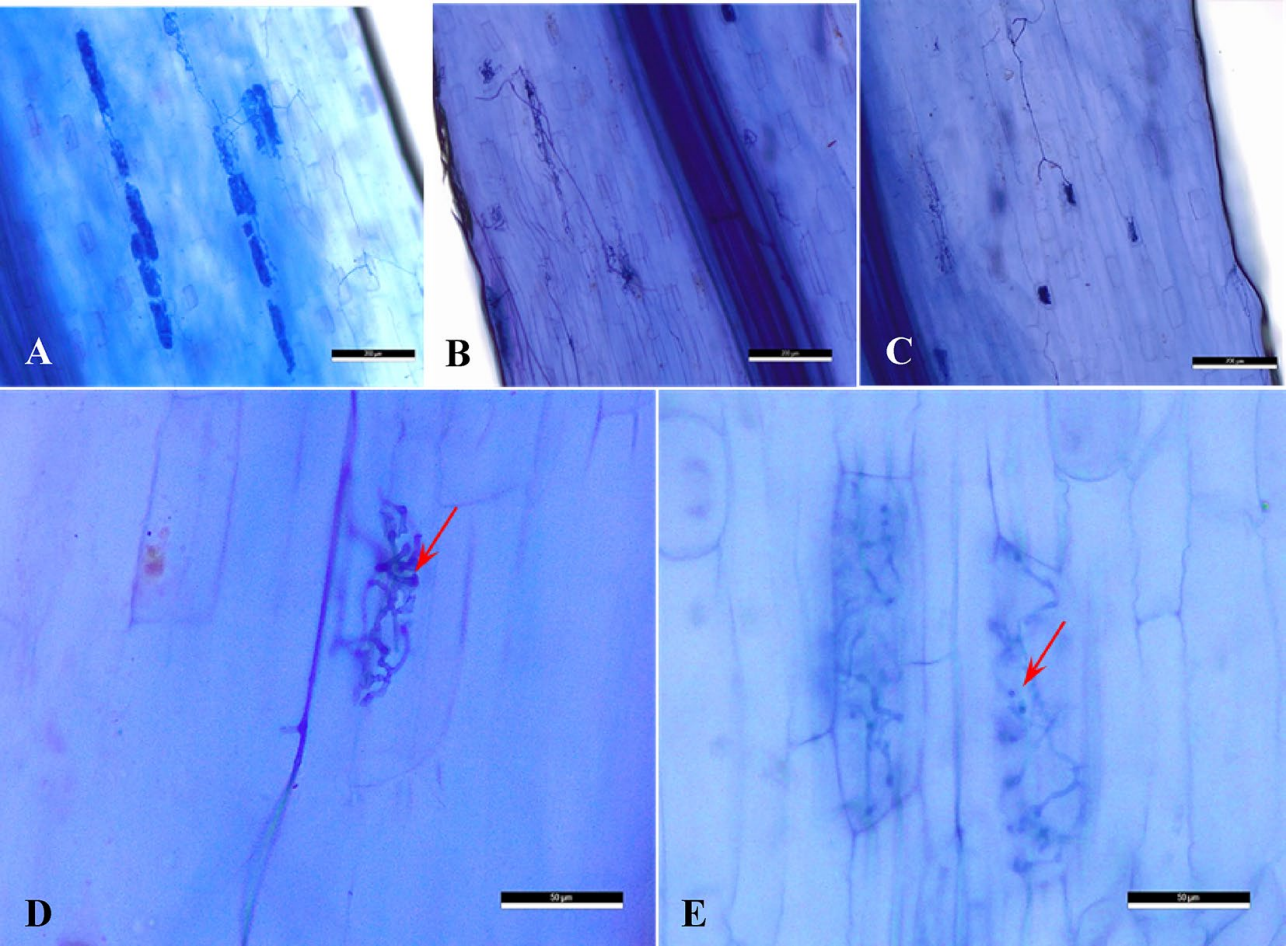
Mycorrhizal infection of onion root inoculated with endomycorrhizal fungi (MF) and irrigated by different concentrations of salty water. **(A)** Mycorrhizal colonization of root at tap water; **(B)** Mycorrhizal colonization of onion root irrigated by 2000 ppm salty water. **(C)** Mycorrhizal colonization of onion root irrigated by 4000 ppm salty water; **(D)** compacted vesicular and Arbuscular structure of onion root irrigated by tap water (arrow); **(E)** loosen vesicular and Arbuscular structure of onion root irrigated by 4000 ppm salty water (arrow).

Data presented in Table 1 and Fig. 1 reveals the infection percentage of endomycorrhizal fungi in the root of onion (*Allium cepa* cv. Giza 20) plants. Generally, the infection percentage decreased by increasing the concentration of salts in the irrigation water. The combined inoculation of *P. putida* and endomycorrhyzae exhibited an increase in the infection percentage compared to single inoculation with endomycorrhyzae only.

**Table 1:** Effect of endomycorrhizal (M), *Pseudomonas putida* (Ps) inoculations individually or combined (MPs) under different levels of salinity on the infection percentage of endomycorrhizal fungi in the root of onion ( *Allium cepa* cv. Giza 20) plants at harvest time.

Treatments		Infection percentage
Control (Tap water)	Control without inoculation	0%
	Inoculated by Ps only	0%
	Inoculated by M only	96%
	Inoculated by MPs	97%
2000 ppm NaCl irrigation water	Control without inoculation	0%
	Inoculated by Ps only	0%
	Inoculated by M only	80%
	Inoculated by MPs	85%
4000 ppm NaCl irrigation water	Control without inoculation	0%
	Inoculated by Ps only	0%
	Inoculated by M only	50%
	Inoculated by MPs	75%

### Effect of biological agents and salinity on morphological characteristics and bulb yield of onion plant

The data presented in (Tables 2, 3) reveals that salt stress negatively affects the onion plant’s growth and production compared to plants not exposed to salt stress, also reveals that plant height, number of leaves, leaf length, leaf diameter, plant fresh weight and plant dry weight showed gradually decreases with increasing salinity levels from 0 ppm (control) to 2000 ppm and 4000 ppm NaCl. *Pseudomonas putida* (Ps) and endomycorrhizal (M) inoculations reduced the negative effects of salinity on onion plants and greatly stimulated all growth parameters whether M and Ps add separately, or MPs combined.

**Table 2:** Effect of Mycorrhizae (M), *Pseudomonas putida* (Ps) inoculations individually or combined (MPs) under different levels of NaCl concentration on some growth parameters of *Allium cepa* cv. Giza 20 plants at 90 days after transplanting.

Treatments	control	M	Ps	MPs	Mean	HSD
	Plant height (cm)					
0 ppm	66.67 ± 4.80^ab^	59.67 ± 4.72^b-d^	63.08 ± 6.58^a-c^	66.93 ± 8.17^ab^	64.09^A^	3.651
2000 ppm	54.75 ± 6.42^d-f^	62.12 ± 8.86^bc^	62.25 ± 5.36^a-c^	69.53 ± 7.71^a^	62.16^A^	
4000 ppm	47.67 ± 2.60^f^	52.25 ± 5.12^ef^	56.75 ± 7.76^c-e^	51.25 ± 4.85^ef^	51.98^B^	
Mean	56.36 ^C^	58.01^BC^	60.69^AB^	62.57^A^		
HSD	4.216					
	Number of leaves/plant					
0 ppm	6.67 ± 1.37^cd^	7.00 ± 0.63^bc^	8.33 ± 1.97^ab^	9.17 ± 1.33^a^	7.79 ^A^	0.771
2000 ppm	4.83 ± 0.75^ef^	7.17 ± 1.17^bc^	7.33 ± 2.25^bc^	7.83 ± 1.60^a-c^	6.79 ^B^	
4000 ppm	3.50 ± 0.84^f^	5.33 ± 1.03^de^	5.17 ± 0.98^de^	5.00 ± 1.10^ef^	4.75 ^C^	
Mean	5.00 ^B^	6.50^A^	6.94^A^	7.33 ^A^		
HSD	0.890					
	Leaf length (cm)					
0 ppm	48.13 ± 2.36^a-c^	45.33 ± 5.68^b-d^	43.67 ± 4.09^c-e^	52.62 ± 8.22^a^	47.44^A^	3.310
2000 ppm	39.67 ± 4.97^d-g^	46.25 ± 8.35^a-d^	45.43 ± 6.60^b-d^	51.33 ± 7.45^ab^	45.67^A^	
4000 ppm	33.50 ± 2.17^g^	38.17 ± 4.25^e-g^	41.00 ± 6.84^d-f^	36.50 ± 3.02^fg^	37.29^B^	
Mean	40.43^B^	43.25^AB^	43.37^AB^	46.82^A^		
HSD	3.822					
	Leaf diameter (cm)					
0 ppm	1.09 ± 0.14^b^	1.15 ± 0.35^b^	2.50 ± 1.31^a^	1.22 ± 0.29^b^	1.49^A^	0.243
2000 ppm	0.88 ± 0.18^b^	1.04 ± 0.15^b^	1.11 ± 0.16^b^	1.10 ± 0.18^b^	1.03^B^	
4000 ppm	0.84 ± 0.06^b^	1.03 ± 0.26^b^	0.98 ± 0.09^b^	0.90 ± 0.09^b^	0.94^B^	
Mean	0.94^B^	1.07^B^	1.53^A^	1.07^B^		
HSD	0.281

**Table 3:** Effect of Mycorrhizae (M), *Pseudomonas putida* (Ps) inoculations individually or combined (MPs) under different levels of NaCl concentration on some growth parameters of *Allium cepa* cv. Giza 20 plants after 150 days for yield.

Treatments	control	M	Ps	MPs		Mean	HSD
	Plant fresh weight (g)						
0 ppm	40.27 ± 8.53^bc^	31.79 ± 9.77^c-e^	42.87 ± 7.62^a-c^	53.55 ± 10.44^a^		42.12^A^	6.058
2000 ppm	24.11 ± 4.21^ef^	36.60 ± 6.97^b-d^	42.41 ± 8.18^a-c^	46.28 ± 7.25^ab^		37.35^A^	
4000 ppm	16.85 ± 1.85^f^	24.81 ± 8.78^d-f^	34.62 ± 9.54^b-e^	24.00 ± 3.17^ef^		25.07^B^	
Mean	27.08^B^	31.07^B^	39.96^A^	41.27^A^			
HSD	6.995						
	Plant dry weight (g)						
0 ppm	5.07 ± 1.20^a-c^	4.26 ± 1.19^b-d^	5.90 ± 1.53^ab^	6.46 ± 1.83^a^		5.42^A^	0.845
2000 ppm	3.64 ± 0.63^cd^	5.06 ± 1.04^a-c^	6.17 ± 1.85^a^	6.38 ± 0.77^a^		5.31^AB^	
4000 ppm	2.96 ± 0.47^d^	4.40 ± 0.97^b-d^	6.43 ± 1.74^a^	4.12 ± 0.60^cd^		4.48^B^	
Mean	3.89^B^	4.57^B^	6.17^A^	5.65^A^			
HSD	0.976						
	Neck thickness (cm)						
0 ppm	4.08 ± 0.20^a^	3.73 ± 0.39^ab^	3.02 ± 1.35^cd^	4.23 ± 0.71^a^		3.77^A^	0.356
2000 ppm	2.97 ± 0.23^cd^	3.85 ± 0.40^a^	4.07 ± 0.84^a^	4.28 ± 0.48^a^		3.79^A^	
4000 ppm	2.32 ± 0.54^d^	3.60 ± 0.71^a-c^	3.12 ± 0.31^bc^	3.10 ± 0.13^bc^		3.03^B^	
Mean	3.12^C^	3.73^AB^	3.40^BC^	3.87^A^			
HSD	0.411						
	Bulb circumference (cm)						
0 ppm	10.43 ± 1.07^ab^	10.55 ± 0.99^ab^	11.03 ± 1.96^a^	10.73 ± 1.27^ab^		10.69^A^	0.829
2000 ppm	9.15 ± 0.99^b-d^	9.68 ± 1.05^a-d^	9.42 ± 1.98^a-d^	9.78 ± 1.60^a-c^		9.51^B^	
4000 ppm	6.67 ± 0.89^e^	8.03 ± 0.92^de^	8.12 ± 1.24^de^	8.73 ± 1.99^cd^		7.89^C^	
Mean	8.75^B^	9.42^AB^	9.52^AB^	9.75^A^			
HSD	0.957						
	Bulb fresh weight (g)						
0 ppm	19.05 ± 4.43^ab^	18.74 ± 4.46^ab^	22.07 ± 3.09^a^	19.70 ± 6.79^ab^		19.89^A^	3.37
2000 ppm	14.10 ± 4.10^b-e^	15.73 ± 4.50^a-d^	16.28 ± 3.24^a-c^	19.17 ± 6.54^ab^		16.32^B^	
4000 ppm	7.99 ± 2.51^e^	9.38 ± 3.26^de^	11.21 ± 4.45^c-e^	14.66 ± 5.03^b-e^		10.81^C^	
Mean	13.71^B^	14.62^AB^	16.52^AB^	17.84 ^A^			
HSD	3.895						
	Bulb dry weight (g)						
0 ppm	2.86 ± 0.76^a-c^	2.90 ± 0.93^a-c^	3.42 ± 1.49^a^	2.86 ± 1.17^a-c^		3.01^A^	
2000 ppm	2.32 ± 0.81^a-e^	2.45 ± 0.69^a-e^	2.60 ± 1.37^a-d^	3.03 ± 1.65^ab^		2.60^A^	
4000 ppm	1.27 ± 0.37^e^	1.61 ± 0.77^de^	1.76 ± 0.83^c-e^	1.99 ± 0.70^b-e^		1.66^B^	0.592
Mean	2.15	2.32	2.59	2.63			
HSD			N.S				

The highest plant height, number of leaves, leaf length, neck thickness and plant fresh weight were shown in MPs treatment across all salinity levels, except only with leaf diameter and plant dry weight which improved by *P. putida* treatment. Also, an insignificant difference was shown between individual treatments of *P. putida* and MPs in all estimated measurements except with leaf diameter and neck thickness. Insignificant differences between M, Ps and MPs were recorded with the number of leaves and leaf length as shown in (Table 2).

When different levels of salinity were compared, insignificant differences were noticed between 0 and 2000 ppm in plant height, leaf length, plant fresh & dry weights, neck thickness and bulb dry weight. Significant reduction was detected when increasing salinity level to 4000 ppm in all growth parameters.

Mycorrhizae (M), Pseudomonas putida (Ps) inoculations individually or combined elevated all plant characterises under different levels of NaCl concentrations. Soil inoculated with MPs under 2000 ppm salinity level recorded the highest value of plant height. While soil inoculated with MPs under 0 ppm salinity level stimulated the increase in number of leaves/ plant and the leaf length/ plant. Soil amended with Ps without salinity increased leaf diameter, bulb circumference, bulb fresh and dry weight, while plant fresh and dry weight and neck thickness recorded the highest increase when the plants cultured in soil supplemented with MPs either in 0 ppm or 2000 ppm salinity level.

Similar decreasing trends in fresh and dry bulb weights were observed with rising salinity levels (Table 3). Inoculation treatments overcome this loss resulting from exposure to salt stress. MPs treatment showed clear stimulation for bulb growth by increasing bulb circumference and its fresh and dry weights. Insignificant differences in bulb dry weight were detected between the three different inoculation treatments.

according to Tukey’s test at p ≤ 0.05, the data show significant differences across treatments if the. means are denoted by different letters. Capital letters are used for means of control, M, Ps, MPs, and salty irrigation water at concentrations of 0, 2000 and 4000 ppm treatments, whereas small letters are used for interactions.

According to Tukey’s test at p ≤ 0.05, the data show significant differences across treatments if the. means are denoted by different letters. Capital letters are used for means of control, M, Ps, MPs, and salty irrigation water at concentrations of 0, 2000 and 4000 ppm treatments, whereas small letters are used for interactions.

When inoculating plants with different biological factors and studying their effect on salinity, the best results were when the plants were treated with the MPs mixture at a salinity level of 2000 ppm, without any significant difference compared to plants not exposed to salt stress.

### Effect of biological agents and salinity on physiological and biochemical characteristics of onion plant

As for biochemical determinations, all determined compounds were affected by exposure to salinity stress but the treatment by biological factors enhanced the plant tolerance under salt stress as shown in Tables 4,5. Salinity reduced leaf chlorophylls and carotenoids concentrations at high levels. Mycorrhizal inoculated plants (M) showed higher chlorophylls than control, but lower than pseudomonas-treated plants (Ps). The mycorrhizae + Pseudomonas (MPs) combination has an additive enhancement in leaf chlorophylls over individual inoculations.Mycorrhizal inoculation (M) helped maintain the carotenoids level highest relative to uninoculated control plants under both non-saline and saline conditions. The highest value for chl A & B were showed with Ps without salinity stress, while the heights value for carotenoids was found at M with 2000 ppm salinity.

**Table 4:** Effect of Mycorrhizae (M), *Pseudomonas putida* (Ps), inoculations individually or combined (MPs) under different levels of NaCl concentration on some physiological parameters of *Allium cepa* cv. Giza 20 plants at 90 days after transplanting.

Treatments	control	M	Ps	MPs	Mean	HSD
	Chl A					
0 ppm	0.22 ± 0.003^de^	0.32 ± 0.032^ab^	0.34 ± 0.006^a^	0.30 ± 0.023^bc^	0.29^A^	0.015
2000 ppm	0.23 ± 0.021^c^	0.24 ± 0.026^c^	0.23 ± 0.002^d^	0.29 ± 0.007^c^	0.25^B^	
4000 ppm	0.14 ± 0.017^f^	0.13 ± 0.008^f^	0.19 ± 0.020^e^	0.23 ± 0.013^d^	0.17^C^	
Mean	0.20^D^	0.23^C^	0.26^B^	0.27^A^		
HSD	0.0172					
	Chl B					
0 ppm	0.15 ± 0.004^bc^	0.17 ± 0.005^b^	0.20 ± 0.009^a^	0.18 ± 0.013^b^	0.17^A^	0.011
2000 ppm	0.13 ± 0.002^de^	0.14 ± 0.015^cd^	0.15 ± 0.010^cd^	0.16 ± 0.010^bc^	0.14^B^	
4000 ppm	0.09 ± 0.008^g^	0.10 ± 0.020^fg^	0.11 ± 0.012^ef^	0.13 ± 0.011^de^	0.11^C^	
Mean	0.12^C^	0.14^B^	0.15^A^	0.16^A^		
HSD	0.0126					
	Carotenoids					
0 ppm	0.027 ± 0.003^ef^	0.038 ± 0.001^b^	0.0304 ± 0.001^c-e^	0.0333 ± 0.001^cd^	0.032^A^	0.0019
2000 ppm	0.025 ± 0.002f	0.044 ± 0.004^a^	0.0296 ± 0.001^cd^	0.0335 ± 0.000^c^	0.033^A^	
4000 ppm	0.027 ± 0.002^ef^	0.016 ± 0.003^g^	0.015 ± 0.003^g^	0.025 ± 0.001f.	0.021^B^	
Mean	0.027^B^	0.033^A^	0.025^B^	0.031^A^		
HSD	0.0026					
	Phenolic compound					
0 ppm	0.96 ± 0.006^g^	1.11 ± 0.072^b-d^	1.17 ± 0.044^b^	0.83 ± 0.010^h^	1.01^B^	0.034
2000 ppm	1.06 ± 0.027^d-f^	1.07 ± 0.003^c-e^	1.04 ± 0.005^d-f^	0.87 ± 0.070^h^	1.01^B^	
4000 ppm	1.38 ± 0.037^a^	1.00 ± 0.052^fg^	1.13 ± 0.028^bc^	1.03 ± 0.042^ef^	1.14^A^	
Mean	1.13^A^	1.06^B^	1.12^A^	0.91^C^		
HSD	0.040					
	Malondialdehyde					
0 ppm	2.28 ± 0.08^c-e^	1.97 ± 0.09^ef^	1.84 ± 0.12^fg^	1.50 ± 0.20^g^	1.90^C^	0.215
2000 ppm	2.38 ± 0.33^b-e^	2.02 ± 0.04^d-f^	2.42 ± 0.06^b-d^	2.17 ± 0.09^d-f^	2.25^B^	
4000 ppm	3.98 ± 0.26^a^	2.74 ± 0.02^b^	2.42 ± 0.08^b-d^	2.67 ± 0.04^bc^	2.95^A^	
Mean	2.88^A^	2.24^B^	2.22^B^	2.11^B^		
HSD	0.248					
	Proline					
0 ppm	89.29 ± 0.40^h^	82.98 ± 4.64^hi^	172.18 ± 4.22^b^	77.53 ± 0.29^i^	105.49^B^	
2000 ppm	179.31 ± 1.38^ab^	181.22 ± 2.57^ab^	100.52 ± 1.48^g^	129.37 ± 7.06^d^	147.61^A^	
4000 ppm	112.01 ± 5.86^f^	162.38 ± 6.24^c^	121.05 ± 8.63^e^	186.61 ± 6.67^a^	145.51^A^	4.16
Mean	126.87^B^	142.19^A^	131.25^B^	131.17^B^		
HSD	4.80					

Phenolic compounds increased under salinity conditions across all treatments. The highest accumulation was seen in plants exposed to 4000 ppm salinity level. Biological agents reduced the accumulation of phenolic compound and MDA in onion plants*.* The reduction in phenolic compounds with *Pseudomonas putida* inoculation (Ps) showed insignificant difference in compared to control plants (Table 4). The MPs treatment achieved the highest reduction in the phenolic compounds. MDA is an indicator of lipid peroxidation from oxidative damage. Inoculated plants with *Pseudomonas putida* and mycorrhizal – *Pseudomonas* mixture showed lower MDA build-up under salt stress relative to controls (Table 4), implying mitigation of membrane damage and oxidative stress by beneficial microbes. The highest values of phenolic compounds and MDA were recorded in uninculated plants exposed to 4000 ppm NaCl.

Increasing salinity from 0 to 2000 ppm NaCl resulted in enhancement of proline content. (Table 4). Mycorrhizal inoculation (M) triggered the maximum increase in proline content relative to control plants.

Plants inoculated with *P. putida* (Ps) and mycorrhizae—*Pseudomonas putida* (MPs) combined treatment also showed higher proline compared to non-inoculated ones, but their degrees reduction were lower than mycorrhizal response with an insignificant difference between both of them. MPs treatment enhanced proline concentration under the highest levels of salinity stress.

The data presented in (Table 5) showed that salinity were increased both of POD and APX antioxidant enzymes activity, these increases were insignificant between both of salinity levels in APX and significant at 2000 ppm level compared to 4000 ppm in POD. Peroxidase and ascorbate peroxidase were elevated by M and Ps treatments. Antioxidant enzymes peroxidase and ascorbate peroxidase were elevated by M treatmentat at 2000 ppm salinity, indicative of activated defense mechanisms. Activities of SOD and PPO followed a similar pattern, increasing with rising salinity. The addition on inoculations either individually or combined together enhanced the activities of SOD and PPO. Individual treatments with M and Ps greatly activate SOD and PPO than MPs MPs combination. Mycorrhizal inoculation modulated antioxidant enzyme levels under salt stress (2000 and 4000 ppm).

**Table 5:** Effect of Mycorrhizae (M), *Pseudomonas putida* (Ps), inoculations individually or combined (MPs) under different levels of NaCl concentration on antioxidant enzymes of *Allium cepa* cv. Giza 20 plants at 90 days after transplanting.

Treatments	control	M	Ps	MPs	Mean	HSD
	POD					
0 ppm	0.70 ± 0.04^i^	1.24 ± 0.13^h^	1.59 ± 0.07^fg^	1.74 ± 0.07^ef^	1.32^C^	0.13
2000 ppm	2.09 ± 0.13^d^	4.99 ± 0.15^a^	3.05 ± 0.20^c^	2.05 ± 0.09^d^	3.05^A^	
4000 ppm	1.40 ± 0.05^gh^	3.94 ± 0.14^b^	2.99 ± 0.35^c^	1.96 ± 0.07^de^	2.57^B^	
Mean	1.40^D^	3.39^A^	2.54^B^	1.92^C^		
HSD	0.14					
	APX					
0 ppm	0.38 ± 0.009^e^	0.30 ± 0.051^f^	0.448 ± 0.008^cd^	0.47 ± 0.017^bc^	0.40^B^	0.024
2000 ppm	0.39 ± 0.021^e^	0.74 ± 0.034^a^	0.45 ± 0.006^c^	0.48 ± 0.007^bc^	0.52^A^	
4000 ppm	0.40 ± 0.045^de^	0.73 ± 0.039^a^	0.51 ± 0.021^b^	0.46 ± 0.028^bc^	0.53^A^	
Mean	0.39^C^	0.59^A^	0.47^B^	0.47^B^		
HSD	0.027					
	SOD					
0 ppm	12.48 ± 0.46^j^	16.84 ± 0.91^i^	25.82 ± 0.76^g^	21.01 ± 1.62^h^	19.04^C^	1.535
2000 ppm	29.93 ± 0.49^f^	46.41 ± 1.09^d^	39.87 ± 1.25^e^	37.48 ± 0.31^e^	38.42^B^	
4000 ppm	44.06 ± 2.87^d^	65.56 ± 2.25^a^	56.84 ± 2.41^b^	52.81 ± 2.14^c^	54.82^A^	
Mean	28.82^D^	42.94^A^	40.84^B^	37.10^C^		
HSD	1.772					
	PPO					
0 ppm	111.20 ± 3.21^k^	176.81 ± 2.59^j^	217.53 ± 4.37^i^	248.41 ± 5.95^h^	188.49^C^	4.91
2000 ppm	258.69 ± 1.88^g^	438.94 ± 9.21^b^	311.12 ± 4.26^f^	268.02 ± 3.83^g^	319.19^B^	
4000 ppm	362.75 ± 4.04^e^	763.58 ± 7.75^a^	399.87 ± 6.81^c^	376.38 ± 5.20^d^	475.64^A^	
Mean	244.21^D^	459.78^A^	309.51^B^	297.60^C^		
HSD	5.67					

According to Tukey’s test at p ≤ 0.05, the data show significant differences across treatments if the.

means are denoted by different letters. Capital letters are used for means of control, M, Ps, MPs, and Salty irrigation water at a concentration of 0, 2000 and 4000 ppm treatments, whereas small letters are used for interactions.

According to Tukey’s test at p ≤ 0.05, the data show significant differences across treatments if the. means are denoted by different letters. Capital letters are used for means of control, M, Ps, MPs, and Salty irrigation water at a concentration of 0, 2000 and 4000 ppm treatments, whereas small letters are used for interactions.

## Discussion

Salinity tolerance is an important issue for agriculture in arid environments, where irrigation water may contain high levels of salts. Onion is considered moderately sensitive to salinity, so high salinity levels in soil can reduce growth, yield and quality parameters^19^. This study investigated the effects of mycorrhizal fungi, Pseudomonas putida, and their combination on onion plants under saline conditions. Mycorrhizal fungi improve phosphorus and nitrogen acquisition^20^. Mycorrhizal inoculation also mitigated the adverse effects of salinity on growth and yield in crops like maize, tomato and pepper^12,21^. On the other hand, the salinity of irrigation water causes a decrease in the infection percentage of mycorrhizae in the roots^22,23^. Actually, the decrease in mycorrhizal infection percentage with increasing salinity observed in our study. However, the combined inoculation of *P. putida* and mycorrhizae (MPs) showed higher infection percentages compared to mycorrhizal inoculation alone, suggesting a synergistic effect between the two microorganisms. According to Hegazi^24^, the infection percentage may be a sign of a plant's tolerance for several types of abiotic stress, particularly salt stress.

The metabolites of *Pseudomonas* spp. like siderophores, organic acids and ACC deaminase can bind toxic ions, provide organic nutrients and lower plant ethylene levels^25^. *Pseudomonas* spp. have been shown to enhance potassium availability, uptake and transport to plants under salinity stress through several mechanisms: solubilizing insoluble soil K minerals via organic acid secretion and lowering pH in the rhizosphere^26^ releasing potassium trapped between clay lattices by extracellular polysaccharides like glucuronic and pyruvic acids^27^, promoting expression and activity of high-affinity K^+^ transporters like HAK5 for enhanced K acquisition even at low external K levels^28^, maintaining integrity of root cell membranes via increasing antioxidant enzyme activities—allowing better functionality of membrane-bound K^+^ channels and transporters under salt stress^7^. Through the above mechanisms, *P. putida* inoculation is expected to increase root K^+^ content, shoot K^+^ levels, higher K^+^/Na^+^ selectivity and improved K^+^ retention in onion leaves subject to salinity and bulb quality compared to uninoculated plants. Research in other crops like wheat and rice found inoculation with *Pseudomonas* spp. helped improve shoot length, dry weight and grain yield under saline conditions compared to uninoculated plants^7^.

Combined application of *P. putida* and mycorrhizal fungi (MPs) may have an additive or synergistic effect on salinity tolerance and can help improve plant growth under saline conditions through several mechanisms via the production of growth-promoting substances, enhancing nutrient uptake especially phosphorus, modulating plant hormone levels, and regulating osmolyte accumulation^29,30^. The MPs metabolites can complement each other to reduce sodium uptake, adjust osmotic balance, maintain nutrition and modulate stress hormones^31^. Our results showed that inoculation with *P. putida*, mycorrhizae and their combination (MPs) improved growth parameters like plant height, bulb diameter/weight and biomass production in onion under 2000 ppm and 4000 ppm salinity compared to uninoculated control. These results were observed in other crops^29,32,33^.

The reduction in photosynthetic pigments with rising salinity indicates impaired functioning of photosynthetic machinery under salt stress^1^. Microbial inoculations like pseudomonas mitigated these effects by mechanisms like enhanced nutrient uptake, antioxidant enzymes^32^. Mycorrhizal symbiosis induced maximum upregulation of leaf pigmentation even without salinity, priming plants for subsequent stress^34^.

The data demonstrates that plant–microbe interactions significantly modulate endogenous accumulation of phenolic compounds under salinity and maintenance of phenols aids antioxidant defense under salt stress. PGPR colonization induces milder adaptive changes in phenolic content in the host plants^35^. While phenols aid antioxidant defense, excessive build-up affects bulb quality during storage^36^. The MPs mixture showed synergism through alternative pathways with restricted stimulation of phenols indicating the involvement of complementary non-phenolic defense pathways in this dual inoculation helping maintain post-harvest attributes.

Mycorrhizae fungi colonization enhances proline synthesis^37^. Proline accumulation contributes to osmotic adjustment and reduces oxidative damage under salt stress^38^ by removing hydroxyl radicals^39^. PGPR modulates proline metabolism for improved osmo-protection in crops under salinity^40^. Our results showed that the combined microbial inoculants (MPs) showed the highest proline accumulation under severe salinity stress, suggesting a synergistic effect in enhancing this osmoprotectant. Combined microbial inoculants employ complementary non-proline pathways for salinity tolerance^32^.

In the present study, *Pseudomonas*, mycorrhizae fungi and the combined microbial inoculants (MPs) showed the lowest MDA accumulation under salinity stress. Malondialdehyde (MDA) is a cytotoxic product of lipid peroxidation and an indicator of free radical-mediated injury and oxidative stress. Salinity induces excessive reactive oxygen species (ROS) generation, resulting in lipid peroxidation and membrane damage. Measurement of MDA levels provides insights into the extent of oxidative damage triggered under salt stress. Beneficial microbes like *Pseudomonas* and mycorrhizae fungi help mitigate oxidative damage by modulating plant antioxidant systems, thereby lowering lipid peroxidation and MDA accumulation^41^ .

In the present study, salinity exposure resulted in significant enhancement in the activities of peroxidase (POD) and ascorbate peroxidase (APX) across all treatments, indicating activation of antioxidant machinery against salt-induced oxidative stress. Mycorrhizal inoculated plants showed a maximal increase in POD and APX activity relative to uninoculated controls under both saline and non-saline conditions, demonstrating fungal-induced modulation of antioxidant metabolism prior to stress onset^21^. While Pseudomonas inoculation also increased POD and APX levels compared to control plants, the degree of enhancement was lower than mycorrhizal response. The mycorrhizae-Pseudomonas combination did not cause any additive change in POD or APX activity over individual inoculations, suggesting the involvement of complementary non-enzymatic pathways for mitigating oxidative damage under stress^42^.

Salinity enhances PPO as part of the oxidative stress defense response in plants^41^. Lower polyphenol oxidase under salt stress indicates inoculum-induced mitigation of oxidative damage^43^. PPO activity changes reflect altered oxidative metabolism during plant–microbe interactions^44^. Mycorrhizal colonization can stimulate plant PPO as an oxidative burst during symbiosis establishment^44^. Combined microbial inoculants can synergistically attenuate salinity-triggered metabolic changes via complementing mechanisms^11^. Polyphenol oxidases play an important role in the biosynthesis and metabolism of phenolic compounds in plants. Some key aspects of PPO-phenolic interrelation: PPO catalyzes the oxidation of phenols as part of plant defense response^45^. Enhanced PPO activity typically parallels rises in endogenous phenolic substrate levels and vice versa^46^.

Superoxide dismutase activity indicates salinity-induced oxidative stress, and elevated SOD levels under salt exposure show activation of antioxidant machinery against oxidative damage^41^. Mycorrhizae fungi can stimulate plant antioxidant enzymes like SOD as a preparative defense response^47^. Mycorrhizae-induced SOD contributes to salinity tolerance. PGPR also provokes SOD induction for managing subsequent abiotic stress^4,7^. Combined inoculants can mitigate salinity effects through non-enzymatic pathways, beyond SOD elevation^34,48^.

## Materials and methods

### Experimental site and biological material

The experiment was carried out in a greenhouse of the Agricultural Botany Department, Faculty of Agriculture. Ain Shams University, Shoubra El-Kheima, Cairo, Egypt, during the years of 2020–2022 on two growing seasons. Cultivated seedlings 60 days old of *Allium cepa* L. cv. Giza 20 that were obtained from Arid Land Agriculture Research Institute, Faculty of Agriculture, Ain Shams University, were used in the experiment as the plant material. Seedlings with similar size were chosen with the permission. All protocols were complied with relevant institutional, national, and international guidelines and legislation.

#### Mycorrhizal inoculant

A mixed culture of endomycorrhizal spores was extracted from the rhizosphere of highly infected endomycorrhizal Barley, grown in the experimental field of the Faculty of Agriculture, Ain Shams University using the wet sieving and decanting technique as described by Gerdemann and Nicolson^49^. Five ml of mycorrhizal spore suspension containing nearby 50 spores ml^−1^ was used as a standard inoculum.

#### Pseudomonas inoculant

*Pseudomonas putida* (EMCCN 1204) with Accession Number (GenBank) OP599903 was obtained from the Microbial Inoculant Centre, Faculty of Agriculture, Ain Shams University. The microbial inoculant was maintained in King’s medium^50^. The microbial densities were adjusted to be 10^10^ cfu ml^−1^_,_

### Treatments

This study was conducted as s a factorial experiment in split plot design, the first factor is salinity stress in three levels; control (tap water), 2000 and 4000 ppm NaCl were provided with irrigation water.

The second factor is two inoculation types endomycorrhizae and *Pseudomonas putida* in four treatments (control, endomycorrhizal inoculant (M), *Pseudomonas* inoculant (Ps) and combination between them (MPs).

#### Inoculation doses

Each seedling received two doses of inoculations, after one week and three weeks of transplanting. Each dose contains 5 ml (that contains 250 spores) of endomycorrhizal suspension and/or 5 ml (10^10^ cfu ml^−1^) of *Pseudomonas putida* at each time. Each application consisted of six pots. There were five plants in each pot (30 cm diameter) as a replicate.

### Estimation of mycorrhizal %

Phillips and Hayman^51^ applied visual observation of fungal colonization in plants to determine the percentage of mycorrhizal root infection. The fraction of mycorrhizal colonization was calculated using the gridline intersect method established by Giovannetti and Mosse^52^.

### Morphological characteristics

The morphological data were recorded as follows: plant height (cm), number of leaves/plant, leaf length (cm), leaf diameter (cm), neck thickness (cm), plant fresh and dry weights (g) through the vegetative growth (after 3 months from transplanting), bulb diameter (cm), bulb fresh and dry weights (g) after 5 months at harvest time.

### Physiological and biochemical analyses

#### Determination of photosynthetic pigments

The photosynthetic pigments (chlorophyll a, chlorophyll b and carotenoids) were evaluated in the 95% ethanolic extract and quantified as mg/g fresh weight (FW) following the method of Sumanta^53^.

#### Determination of total soluble phenolic compounds

The amount of total soluble phenolic compounds was measured using the Folin-Ciocalteu colorimetric method as described by Shahidi and Naczk^54^. Gallic acid was utilized as a standard reference material and the concentration of total phenols was expressed as mg/g FW.

#### Determination of proline concentration

The amount of proline was measured using a color change technique developed by Troll and Lindsley^55^ and later adapted by Peters^56^. This involved reacting proline with ninhydrin and measuring the intensity of the color produced at 515 nm. The final proline concentration was reported as μg proline /g of fresh plant material.

#### Determination of lipid peroxidation

The extent of lipid peroxidation was assessed by measuring malondialdehyde (MDA), a product of lipid peroxidation, using the thiobarbituric acid (TBA) test as described by Heath and Packer^57^. In this method, MDA reacts with TBA to form a colored complex that can be measured spectrophotometrically. The supernatant was analyzed by recording the absorbance at 535 nm. The MDA concentration expressed as nmol /g fresh weight.

#### Assay of guaiacol peroxidase (G-POD) activity

The activity of G-POD (E.C 1.11.1.7) was measured following the procedure described by Hammer Schmidt^58^. It was determined by monitoring the change in absorbance at 470 nm.

#### Assay of ascorbate peroxidase (APX) activity

The activity of APX (EC 1.11.1.11) was determined using the method described by Nakano and Asada^59^. This involved monitoring the reduction in absorbance at 290 nm over 3 min as ascorbate was oxidized. One unit of APX activity was defined as the quantity of enzyme required to oxidize 1 micromole of ascorbate per minute.

#### Assay of superoxide dismutase (SOD) activity

The measurement of SOD (EC 1.15.1.1) activity relied on the technique outlined by Beyer and Fridovich^60^. One unit of SOD activity was characterized as the quantity of enzyme necessary to produce 50% suppression in the rate of nitro blue tetrazolium reduction at 560 nm.

#### Assay of polyphenol oxidase (PPO) activity

The activity of PPO (EC 1.14.18.1) was determined as described by Oktay^61^. The absorbance at 420 nm was measured at the start and after 1 min. One unit of PPO activity was defined as the quantity of enzyme causing an increase in absorbance of 0.001 per minute at 42 nm.

All enzymes activity was conveyed as units per milligram of protein.

### Statistical analysis

SAS software^62^ was utilized to carry out a two-way analysis of variance (ANOVA). Five replicates were used to calculate the mean values ± SD, and the significant difference between the means was employed by Tukey’s Studentized Range (HSD) at* p* ≤ 0.05.

## Conclusion

This study demonstrates the significant potential of using microbial inoculants, specifically endomycorrhizal fungi (M) and *Pseudomonas putida* (Ps), to enhance salt tolerance in onion crops. The combined application of these beneficial microorganisms (MPs) proved particularly effective in mitigating the adverse effects of salinity stress on onion growth, leaf pigments, bulb development and activated antioxidant defenses. The results highlight the potential of microbial inoculants as an eco-friendly and sustainable approach to improving onion productivity in saline conditions. The synergistic effects observed with combined inoculation (MPs) suggest that this approach could be particularly promising for agricultural applications.

In future research directions, field trials to validate the greenhouse results and assess the long-term effects of these inoculants under varying environmental conditions. Assessment of the impact of these inoculants on onion bulb quality and post-harvest characteristics. Evaluation of the economic feasibility and environmental impact of large-scale application of these microbial inoculants. Investigation of the potential of these inoculants to mitigate other abiotic stresses in onion and related crops.

## Data Availability

The authors can confirm that all relevant data are included in the article.

## References

[1] Munns, R. & Tester, M. Mechanisms of salinity tolerance. *Annu. Rev. Plant Biol.* **59**, 651–681. 10.1146/annurev.arplant.59.032607.092911 (2008). 18444910 10.1146/annurev.arplant.59.032607.092911

[2] Maas, E. V. Crop salt tolerance. In *Agricultural salinity assessment and management. american society of civil engineers* (ed. Tanji, K. K.) 262–304 (New York, 1990).

[3] Garcia, G., Garcia, M. & Ramirez, H. Performance in seven Allium cepa L cultivars at different salt stress levels. *Bioagro.* **27**, 93–102 (2015).

[4] Sharma, P. C., Mishra, B., Singh, R. K., Singh, Y. P. & Tyagi, N. K. Adaptability of onion (*Allium cepa*) genotypes to alkali and salinity stresses. *Indian J. Agric. Sci.* **70**, 674–678 (2001).

[5] Bhattacharyya, P. N. & Jha, D. K. Plant growth-promoting rhizobacteria (PGPR): Emergence in agriculture. *World J. Microbiol. Biotechnol.* **28**, 1327–1350. 10.1007/s11274-011-0979-9 (2012). 22805914 10.1007/s11274-011-0979-9

[6] Praveen Kumar, G. *et al.**In vitro* screening for abiotic stress tolerance in potent biocontrol and plant growth promoting strains of pseudomonas and bacillus spp. *Int J Bacteriol*10.1155/2014/195946 (2014). 26904731 10.1155/2014/195946 PMC4745462

[7] Jha, Y., Subramanian, R. B. & Patel, S. Combination of endophytic and rhizospheric plant growth promoting rhizobacteria in *Oryza sativa* shows higher accumulation of osmoprotectant against saline stress. *Acta Physiol. Plant* **33**, 797–802. 10.1007/s11738-010-0604-9 (2011). 10.1007/s11738-010-0604-9

[8] Abulfaraj, A. A. & Jalal, R. S. Use of plant growth-promoting bacteria to enhance salinity stress in soybean (Glycine max L.) plants. *Saudi J Biol Sci.* **28**(7), 3823–3834. 10.1016/j.sjbs.2021.03.053 (2021). 34220237 10.1016/j.sjbs.2021.03.053 PMC8241701

[9] Cantrell, I. C. & Linderman, R. G. Preinoculation of lettuce and onion with VA mycorrhizal fungi reduces deleterious effects of soil salinity. *Plant Soil* **233**, 269–281. 10.1023/A:1010564013601 (2001). 10.1023/A:1010564013601

[10] López-Ráez, J. A. *et al.* Hormonal and transcriptional profiles highlight common and differential host responses to arbuscular mycorrhizal fungi and the regulation of the oxylipin pathway. *J. Exp. Bot.* **61**, 2589–2601. 10.1093/jxb/erq089 (2010). 20378666 10.1093/jxb/erq089 PMC2882257

[11] Vurukonda, S. S. K. P., Vardharajula, S., Shrivastava, M. & Sk, Z. A Enhancement of drought stress tolerance in crops by plant growth promoting rhizobacteria. *Microbiol. Res.* **184**, 13–24. 10.1016/j.micres.2015.12.003 (2016). 26856449 10.1016/j.micres.2015.12.003

[12] Estrada, B., Aroca, R., Maathuis, F. J. M., Barea, J. M. & Ruiz-Lozano, J. M. Arbuscular mycorrhizal fungi native from a mediterranean saline area enhance maize tolerance to salinity through improved ion homeostasis. *Plant Cell Environ.* **36**, 1771–1782. 10.1111/pce.12082 (2013). 23421735 10.1111/pce.12082

[13] Ruiz-Lozano, J. M., Porcel, R., Azcón, C. & Aroca, R. Regulation by arbuscular mycorrhizae of the integrated physiological response to salinity in plants: New challenges in physiological and molecular studies. *J. Exp. Bot.* **63**, 4033–4044. 10.1093/jxb/ers126 (2012). 22553287 10.1093/jxb/ers126

[14] Porcel, R., Aroca, R., Azcon, R. & Ruiz-Lozano, J. M. Regulation of cation transporter genes by the arbuscular mycorrhizal symbiosis in rice plants subjected to salinity suggests improved salt tolerance due to reduced Na^+^ root-to-shoot distribution. *Mycorrhiza* **26**, 673–684. 10.1007/s00572-016-0704-5 (2016). 27113587 10.1007/s00572-016-0704-5

[15] Porcel, R., Aroca, R. & Ruiz-Lozano, J. M. Salinity stress alleviation using arbuscular mycorrhizal fungi a review. *Agron. Sustain. Dev.* **32**, 181–200. 10.1007/s13593-011-0029-x (2012). 10.1007/s13593-011-0029-x

[16] Nadeem, S. M., Ahmad, M., Zahir, Z. A., Javaid, A. & Ashraf, M. The role of mycorrhizae and plant growth promoting rhizobacteria (PGPR) in improving crop productivity under stressful environments. *Biotechnol. Adv.* **32**, 429–448. 10.1016/j.biotechadv.2013.12.005 (2014). 24380797 10.1016/j.biotechadv.2013.12.005

[17] Kumar, Y. V. *et al.* Traversing arbuscular mycorrhizal fungi and *Pseudomonas fluorescens* for carrot production under salinity. *Saudi J. Biol. Sci.* **28**, 4217–4223. 10.1016/j.sjbs.2021.06.025 (2021). 34354402 10.1016/j.sjbs.2021.06.025 PMC8325001

[18] Shinde, S., Zerbs, S. & Collart, F. R. *Pseudomonas fluorescens* increases mycorrhization and modulates expression of antifungal defense response genes in roots of aspen seedlings. *BMC Plant Biol.* **19**, 4. 10.1186/s12870-018-1610-0 (2019). 30606121 10.1186/s12870-018-1610-0 PMC6318872

[19] Alam, M. A. *et al.* Performance valuation of onion (*Allium cepa L*) genotypes under different levels of salinity for the development of cultivars suitable for saline regions. *Front. Plant Sci.*10.3389/fpls.2023.1154051 (2023). 37063224 10.3389/fpls.2023.1154051 PMC10102481

[20] Nouri, E., Breuillin-Sessoms, F., Feller, U. & Reinhardt, D. Phosphorus and nitrogen regulate arbuscular mycorrhizal symbiosis in *Petunia hybrida*. *PLoS One.* **9**, e90841. 10.1371/journal.pone.0090841 (2015). 10.1371/journal.pone.0090841 PMC3946601 24608923

[21] Abdel Latef, A. A. H. & Chaoxing, H. Effect of arbuscular mycorrhizal fungi on growth, mineral nutrition, antioxidant enzymes activity and fruit yield of tomato grown under salinity stress. *Sci. Hortic.* **127**, 228–233. 10.1016/j.scienta.2010.09.020 (2011). 10.1016/j.scienta.2010.09.020

[22] Manaf, H. H. & Zayed, M. S. Productivity of cowpea as affected by salt stress in presence of endomycorrhizae and *Pseudomonas fluorescens*. *Ann. Agric. Sci.* **60**, 219–226. 10.1016/j.aoas.2015.10.013 (2015). 10.1016/j.aoas.2015.10.013

[23] Zayed, M. S., Hegazi, G. A., Salem, H. M. & Ibrahim, W. M. Role of endomycorrhizae and *Pseudomonas fluorescens* on the acclimatization of micropropagated *Stevia rebaudiana* Bert. plantlets. *Afr. J. Plant Sci.* **11**, 38–47. 10.5897/AJPS2016.1494 (2017). 10.5897/AJPS2016.1494

[24] Hegazi, G. A., Zayed, M. S., Salem, H. M. & Ibrahim, W. M. Effect of explant type and sequential subcultures on *in vitro* multiple shoots formation of jojoba. *J. Appl. Environ. Biol. Sci.* **4**, 214–222 (2014).

[25] Sagar, A. *et al.* plant growth promoting rhizobacteria, arbuscular mycorrhizal fungi and their synergistic interactions to counteract the negative effects of saline soil on agriculture: Key macromolecules and mechanisms. *Microorganisms* **9**, 1491. 10.3390/microorganisms9071491 (2021). 34361927 10.3390/microorganisms9071491 PMC8307984

[26] Nawaz, A. *et al.* Contribution of potassium solubilizing bacteria in improved potassium assimilation and cytosolic K+/Na+ ratio in rice (*Oryza sativa L.)* under saline-sodic conditions. *Front. Microbiol.* **14**, 1196024. 10.3389/fmicb.2023.1196024 (2023). 37711698 10.3389/fmicb.2023.1196024 PMC10497963

[27] Kang, S. M. *et al.* Gibberellin secreting rhizobacterium, *Pseudomonas putida* H-2-3 modulates the hormonal and stress physiology of soybean to improve the plant growth under saline and drought conditions. *Plant Physiol. Biochem.* **84**, 115–124. 10.1016/j.plaphy.2014.09.001 (2014). 25270162 10.1016/j.plaphy.2014.09.001

[28] Haro, R. & Benito, B. The role of soil fungi in K^+^ plant nutrition. *Int. J. Mol. Sci.* **20**, 3169. 10.3390/ijms20133169 (2019). 31261721 10.3390/ijms20133169 PMC6651076

[29] Zuccarini, P. & Okurowska, P. Effects of mycorrhizal colonization and fertilization on growth and photosynthesis of sweet basil under salt stress. *J. Plant Nutr.* **31**, 497–513. 10.1080/01904160801895027 (2008). 10.1080/01904160801895027

[30] Zaller, J. G. & Kopke, U. Effects of traditional and biodynamic farmyard manure amendment on yields, soil chemical, biochemical and biological properties in a long-term field experiment. *Biol. Fertil. Soils* **40**, 222–229. 10.1007/s00374-004-0772-0 (2004). 10.1007/s00374-004-0772-0

[31] Moreira, H., Pereira, S. I. A., Vega, A., Castro, P. M. L. & Marques, A. P. G. C. Synergistic effects of arbuscular mycorrhizal fungi and plant growth-promoting bacteria benefit maize growth under increasing soil salinity. *J. Environ. Manage.* **257**, 109982. 10.1016/j.jenvman.2019.109982 (2020). 31868642 10.1016/j.jenvman.2019.109982

[32] Kohler, J., Caravaca, F. & Roldan, A. An AM fungus and a PGPR intensify the adverse effects of salinity on the stability of rhizosphere soil aggregates of *Lactuca sativa*. *Soil Biol. Biochem.* **42**, 429–434. 10.1016/j.soilbio.2009.11.021 (2010). 10.1016/j.soilbio.2009.11.021

[33] Norouzinia, F., Ansari, M. H., Aminpanah, H. & Firozi, S. Alleviation of soil salinity on physiological and agronomic traits of rice cultivars using arbuscular mycorrhizal fungi and pseudomonas strains under field conditions. *Rev. Agric. Neotrop.* **7**, 25–42. 10.32404/rean.v7i1.4042 (2020). 10.32404/rean.v7i1.4042

[34] Sheng, M. *et al.* Influence of arbuscular mycorrhizae on photosynthesis and water status of maize plants under salt stress. *Mycorrhiza* **18**, 287–296. 10.1007/s00572-008-0180-7 (2008). 18584217 10.1007/s00572-008-0180-7

[35] Korenblum, E. & Aharoni, A. Phytobiome metabolism: Beneficial soil microbes steer crop plants’ secondary metabolism. *Pest Manag. Sci.* **75**, 2378–2384. 10.1002/ps.5440 (2019). 30973666 10.1002/ps.5440

[36] Osorio-Esquivel, O., Álvarez, V. B., Dorantes-Álvarez, L. & Giusti, M. M. Phenolics, betacyanins and antioxidant activity in *Opuntia joconostle* fruits. *Food. Res. Int.* **44**, 2160–2168. 10.1016/j.foodres.2011.02.011 (2011). 10.1016/j.foodres.2011.02.011

[37] Hajiboland, R., Aliasgharzadeh, N. & Laiegh, S. F. Colonization with arbuscular mycorrhizal fungi improves salinity tolerance of tomato (Solanum lycopersicum L.) plants. *Plant Soil* **331**, 313–327. 10.1007/s11104-009-0255-z (2010). 10.1007/s11104-009-0255-z

[38] Hayat, S. *et al.* Role of proline under changing environments. *Plant Signal Behav.* **7**, 1456–1466. 10.4161/psb.21949 (2012). 22951402 10.4161/psb.21949 PMC3548871

[39] Rhodes, D., Nadolska-Orczyk, A. & Rich, P. Salinity, osmolytes and compatible solutes. In *Salinity: Environment - Plants - Molecules)* (eds Läuchli, A. & Lüttge, U.) (Springer, 2004).

[40] Kasotia, A., Varma, A. & Choudhary, D. K. Pseudomonas-mediated mitigation of salt stress and growth promotion in glycine max. *Agric. Res.* **4**, 31–41. 10.1007/s40003-014-0139-1 (2015). 10.1007/s40003-014-0139-1

[41] Tuna, A. L., Kaya, C., Dikilitas, M. & Higgs, D. The combined effects of gibberellic acid and salinity on some antioxidant enzyme activities, plant growth parameters and nutritional status in maize plants. *Environ. Exp. Bot.* **62**, 1–9. 10.1016/j.envexpbot.2007.06.007 (2008). 10.1016/j.envexpbot.2007.06.007

[42] Kohler, J., Hernández, J. A., Caravaca, F. & Roldán, A. Plant-growth-promoting rhizobacteria and arbuscular mycorrhizal fungi modify alleviation biochemical mechanisms in water-stressed plants. *Funct. Plant Biol.* **35**, 141–151. 10.1071/FP07218 (2008). 32688765 10.1071/FP07218

[43] Kesawat, M. S. *et al.* Regulation of reactive oxygen species during salt stress in plants and their crosstalk with other signaling molecules-current perspectives and future directions. *Plants (Basel).* **12**, 864. 10.3390/plants12040864 (2023). 36840211 10.3390/plants12040864 PMC9964777

[44] Mayer, Z., Duc, N. H., Sasvári, Z. & Posta, K. How arbuscular mycorrhizal fungi influence the defense system of sunflower during different abiotic stresses. *Acta Biol. Hung.* **68**, 376–387. 10.1556/018.68.2017.4.4 (2017). 29262715 10.1556/018.68.2017.4.4

[45] Thipyapong, P., Stout, M. J. & Attajarusit, J. Functional analysis of polyphenol oxidases by antisense/sense technology. *Molecules* **12**, 1569–1595. 10.3390/12081569 (2007). 17960074 10.3390/12081569 PMC6149088

[46] Sikora, M. *et al.* Biochemical properties of polyphenol oxidases from ready-to-eat lentil (Lens culinaris Medik.) sprouts and factors affecting their activities: A search for potent tools limiting enzymatic browning. *Foods* **8**, 154. 10.3390/foods8050154 (2019). 31067803 10.3390/foods8050154 PMC6560442

[47] Hashem, A., Abd-Allah, E. F., Alqarawi, A. A., Aldubise, A. & Egamberdieva, D. Arbuscular mycorrhizal fungi enhances salinity tolerance of *Panicum turgidum* forssk by altering photosynthetic and antioxidant pathways. *J. Plant Interact.* **10**, 230–242. 10.1080/17429145.2015.1052025 (2015). 10.1080/17429145.2015.1052025

[48] Ramadoss, D., Lakkineni, V. K. & Bose, P. Mitigation of salt stress in wheat seedlings by halotolerant bacteria isolated from saline habitats. *SpringerPlus* **2**, 6. 10.1186/2193-1801-2-6 (2013). 23449812 10.1186/2193-1801-2-6 PMC3579424

[49] Gerdemann, J. W. & Nicolson, T. H. Spores of mycorrhizal endogone species extracted from soil by wet sieving and decanting. *Trans. Br. Mycol. Soc.* **46**, 235–244. 10.1016/S0007-1536(63)80079-0 (1963). 10.1016/S0007-1536(63)80079-0

[50] King, E. D., Ward, M. K. & Raney, D. E. Two simple media for the demonstration of pyocyanin and fluorescin. *J. Lab. Clin. Med.* **44**, 301–307. 10.5555/uri:pii:002221435490222X (1954). 13184240 10.5555/uri:pii:002221435490222X

[51] Phillips, J. M. & Hayman, D. A. Improved procedures for clearing roots and staining parasitic and vesicular-arbuscular mycorrhizal fungi for rapid assessment of infection. *Trans. Br. Mycol. Soc.* **55**, 158–161. 10.1016/S0007-1536(70)80110-3 (1970). 10.1016/S0007-1536(70)80110-3

[52] Giovannetti, M. & Mosse, B. An evaluation of techniques for measuring vesicular arbuscular mycorrhizal infection in roots. *New Phytol.* **84**, 489–500. 10.1111/j.1469-8137.1980.tb04556.x (1980). 10.1111/j.1469-8137.1980.tb04556.x

[53] Sumanta, N., Haque, C. I., Nishika, J. & Suprakash, R. Spectrophotometric analysis of chlorophylls and carotenoids from commonly grown fern species by using various extracting solvents. *Res. J. Chem. Sci.* **4**, 63–69. 10.1055/s-0033-1340072 (2014). 10.1055/s-0033-1340072

[54] Shahidi, F. & Naczk, M. *Food Phenolics: Sources, Chemistry, Effects and Applications. 287–293* (Technomic Publishind Company Inc, 1995).

[55] Troll, W. & Lindsley, J. A photometric method for the determination of proline. *J. Biol. Chem.* **215**, 655–660. 10.1016/S0021-9258(18)65988-5 (1955). 13242563 10.1016/S0021-9258(18)65988-5

[56] Peters, W., Beck, E., Piepenbrock, M., Lenz, B. & Schmitt, J. M. Cytokinine as a negative effector of phosphoenolpyruvate carboxylase induction in *Mesembryanthemum crystallinum*. *J. Plant Physiol.* **151**, 362–367. 10.1016/S0176-1617(97)80266-0 (1997). 10.1016/S0176-1617(97)80266-0

[57] Heath, R. L. & Packer, L. Photoperoxidation in isolated chloroplasts. 1. Kinetics and stoichiometry of fatty acids peroxidation. *Arch. Biochem. Biophys* **125**, 189–198. 10.1016/0003-9861(68)90654-1 (1968). 5655425 10.1016/0003-9861(68)90654-1

[58] Hammer Schmidt, R., Nuckles, E. M. & Kuc, J. Association of enhanced peroxidase activity with induced systemic resistance of cucumber to *Colletotrichum lagenarium*. *Physiol. Plant* **20**, 73–82. 10.1016/0048-4059(82)90025-X (1982). 10.1016/0048-4059(82)90025-X

[59] Nakano, Y. & Asada, K. Hydrogen peroxide is scavenged by ascorbate specific peroxidase in spinach chlroplasts. *Plant Cell Physiol.* **22**, 867–880. 10.1093/oxfordjournals.pcp.a076232 (1981). 10.1093/oxfordjournals.pcp.a076232

[60] Beyer, J. W. F. & Fridovich, I. Assaying for superoxide dismutase activity: some large consequences of minor changes in condition. *Anal Biochem.* **161**, 559–566. 10.1016/0003-2697(87)90489-1 (1987). 3034103 10.1016/0003-2697(87)90489-1

[61] Oktay, M., Küfrevioğlu, I., Kocacalıskan, I. & Sakiroğlu, H. Polyphenol oxidase from Amasya apple. *J. Food Sci.* **60**, 495–499. 10.1111/j.1365-2621.1995.tb09810.x (1995). 10.1111/j.1365-2621.1995.tb09810.x

[62] SAS Statistical analysis system, SAS User's Guide: Statistics. (SAS Institute Inc., Editor, Cary, NC 2003).

